# Evaluation of a Salt-Reduction Consumer Awareness Campaign Targeted at Parents Residing in the State of Victoria, Australia

**DOI:** 10.3390/nu15040991

**Published:** 2023-02-16

**Authors:** Carley A. Grimes, Kristy A. Bolton, Karen Lim, Durreajam Khokhar, Joseph Alvin Santos, Kathy Trieu, Claire Margerison, Jenny Reimers, Sian Armstrong, Bruce Bolam, Emalie Rosewarne, Elizabeth K. Dunford, Stephen Jan, Mark Woodward, Bruce Neal, Caryl Nowson, Jacqui Webster

**Affiliations:** 1 Institute for Physical Activity and Nutrition, Deakin University, Geelong 3216, Australia; 2 School of Exercise and Nutrition Sciences, Deakin University, Geelong 3216, Australia; 3 The George Institute for Global Health, University of New South Wales, Sydney 2050, Australia; 4 Victorian Health Promotion Foundation (VicHealth), Melbourne 3003, Australia; 5 Heart Foundation, Melbourne 3008, Australia; 6 Loddon Mallee Public Health Unit, Bendigo 3550, Australia; 7 Department of Nutrition, The University of North Carolina at Chapel Hill, Chapel Hill, NC 27516, USA; 8 The George Institute for Global Health, School of Public Health, Imperial College London, London SW7 2BX, UK

**Keywords:** salt, sodium, knowledge, attitude, behavior, parents, Australia

## Abstract

From 2015 to 2020 a state-wide salt-reduction initiative was launched in Victoria, Australia, including an awareness campaign focused on parents with children <18 years of age. To evaluate the impact of the campaign on salt-related knowledge, attitudes and behaviors (KABs) we have assessed trends in salt-related KAB pre- and post-delivery of the campaign in parents, as well as within the wider adult population. Cross-sectional surveys of adults aged 18–65 years were undertaken pre- (2015: *n* = 821 parents; *n* = 1527 general sample) and post-campaign (2019: *n* = 935 parents; *n* = 1747 general sample). KABs were assessed via an online survey. Data were analyzed with regression models and adjusted for covariates. Among parents, around one-quarter of salt-related KABs shifted in a positive direction, but changes were small: there was a 6% (95% CI 2, 11%) increase in the percentage who knew the main source of salt in the diet and reductions in the percentage who reported placing a salt shaker on the table (−8% (95%CI −12, −3)) and that their child added salt at the table (−5% (95% −9, −0.2)). Among the wider adult sample, even fewer shifts in KAB were observed, with some behaviors worsening at follow-up. These findings indicate that this consumer awareness campaign had minimum impact.

## 1. Introduction

A diet high in sodium contributes to high blood pressure and increases the risk of cardiovascular disease (CVD) [1]. The 2017 Global Burden of Disease study found that a high sodium intake was a leading dietary risk factor for disease burden, accounting for 3 million deaths and 70 million disability adjusted life-years (DALYs) [2]. Most sodium is consumed in the form of salt (sodium chloride) which can be added during the manufacture of processed foods, to foods prepared within the food service (restaurants/cafes) or by the consumer during cooking or at the table. Globally, it is estimated that adults consume on average 10.1 g of salt per day [3] and in response to high intakes, many countries have implemented national salt-reduction initiatives [4]. These strategies align with the World Health Organization’s target to reduce global population salt intake by 30% by 2025 to reduce mortality and morbidity from non-communicable diseases [5]. While past salt-reduction initiatives have varied in terms of strategies and scale of implementation, a common feature across many has been the inclusion of consumer awareness campaigns. These are designed to educate the public on the health risks associated with a high salt diet as well as behavioral changes that can be made to help lower the amount of salt in the diet (e.g., reading food labels to pick lower salt foods and avoiding table and cooking salt [6]). A notable example is the United Kingdom’s Food Standards Agency salt-reduction strategy which was implemented between 2004 and 2009. The success of this campaign in shifting the population’s salt-related knowledge, attitudes and behaviors [7] has made it a model for other countries wishing to design and implement salt-related awareness campaigns.

Similar to the many other countries, a high salt diet is a problem in Australia, with adults consuming 9.6 g of salt per day [8], almost double the recommended limit of 5 g per day [9,10], and three-quarters of children exceeding recommendations [11,12]. Therefore, there is a need for strategies to reduce Australians’ salt intake and in turn, protect cardiovascular health. Although the Australian Government has adopted the WHO’s target of reducing population salt intake by 30% by 2025 [13,14], no coordinated national strategy or action plan has been established [4,15]. As such, in 2014 VicHealth (the Victorian Health Promotion Foundation in the Australian state of Victoria), in collaboration with peak public health organizations, embarked on a state-wide initiative to reduce the average salt intake of adults and children by one gram per day by 2020 [16,17]. The multi-faceted intervention was developed and overseen by the Victorian Salt Reduction Partnership, a group of stakeholders from health-related non-governmental organizations, the state government and the academic sector. Intervention strategies were informed by previous state and community-level salt-reduction interventions [18,19] and centered around five key action areas—raising consumer awareness; generating public debate; strategic partnership; engaging food industry; and advocacy and policy strengthening [20,21]. Central to the intervention was a consumer awareness campaign delivered during 2016–2019 [16,20] that aimed to support consumers in reducing their salt intake and encourage community support for reduced-salt foods. Specifically, the campaign sought to raise awareness about salt in the diet (e.g., knowledge of high intakes in the community, associated health risks, hidden salt in packaged foods) and shift attitudes surrounding the importance of reducing salt intake, which in turn would lead to the adoption of salt-lowering behaviors (e.g., reading food labels to pick lower salt options and using less cooking and table salt) [16]. The primary target audience was household food providers with children; however, messages were also intended to reach the broader Victorian adult population. For example, while paid campaign advertisements were specifically shown on social media feeds of parents, salt awareness stories also appeared on wider-reaching breakfast TV programs and radio [20]. 

Therefore, to evaluate the impact of the consumer awareness campaign on salt-related knowledge, attitudes and behaviors (KABs) within the Victorian population we have assessed trends in salt-related KAB pre- and post-delivery of the campaign in two segments of the population: i) the target market of the campaign (parents of children <18 years of age) and ii) the wider adult population (excluding parents of children <18 years of age). This study sits within a comprehensive evaluation of the Victorian Salt Reduction Partnership salt-reduction intervention [16,20,21,22].

## 2. Materials and Methods

### 2.1. Study Design and Recruitment of Participants

This was a repeated cross-sectional survey of Victorian adults aged 18–65 years completed before (September–November 2015) [23] and after the salt awareness campaign (April–June 2019). Recruitment quotas were based on age and sex groups reflecting the Victorian population [24]. Participants >65 years of age were excluded as future salt-related public awareness campaigns would primarily target adults below this age. All participants provided consent to participate and ethics approval was granted by the Deakin University Human Ethics Advisory Group (Project No. HEAG-H 83_2015 and HEAG-H 71_2016). To help capture a representative sample of the population, three strategies were used to recruit participants: (i) an intercept survey within shopping centers, (ii) a Facebook advertisement campaign and (iii) an online consumer research panel (Lightspeed Research). Shopping centers were located in Greater Melbourne (three sites) and Geelong (one site) and included two centers from a higher socioeconomic area and two centers from a lower socioeconomic area [23]. The same centers were used at each time point and data were primarily collected Monday to Saturday, 9:00 a.m. to 5:00 p.m.; however, some Sundays and late night shopping hours were included. Research staff invited passing-by shoppers to participate in the study. Shoppers aged <18 years and >65 years were excluded (*n* = 156 at baseline and *n* = 210 at follow-up). Facebook recruitment consisted of a ‘clicks to website’ advertisement that invited users who were Victorian residents aged 18–64 years to complete the online survey. A commercial research company (Lightspeed Research) was enlisted to invite its panel members to complete the online survey in exchange for in-company reward points which are redeemable for monetary payments. 

### 2.2. Survey Instrument

Participants were asked to independently complete an online 32 item survey assessing demographic characteristics, KABs related to salt intake and their recognition of the salt-reduction consumer awareness campaign [23]. This analysis includes survey items assessing salt-related knowledge (5 questions), attitudes (3 questions) and behaviors (5 questions) that were relevant to the evaluation of the salt awareness campaign (Supplementary File S1). Participants that identified as being a parent or caregiver for children under 18 years of age, hereafter referred to as parents, were asked an additional seven questions related to children’s salt intake (2 knowledge, 2 attitudes and 3 behaviors). Questions were modeled on those used in previous salt surveys [25,26,27,28,29,30,31,32,33,34,35,36,37,38]. Demographic characteristics assessed included age, sex, educational attainment (low: those with some or no level of high school education; mid: those with a technical/trade certificate or diploma; high: those with a university/tertiary qualification), residential postcode, country of birth, language spoken at home, responsibility for household grocery shopping, self-reported weight and height, diagnosis of a chronic condition, use of antihypertensive medication and if they had previously received advice from a health professional to limit salt intake. Participants were grouped into weight categories according to calculated body mass index (BMI).

### 2.3. Power Calculations

Pre-campaign sample size calculations were based on the expected proportion (0.7) [31] of participants (regardless of parent/caregiver status) who would respond yes to key salt KABs questions. Assuming a design effect of 1.5 and reporting estimates across four age groups, a sample size of 1936 was estimated to produce a margin of error of no more than ± 5% points for a survey response at the 95% confidence level [38]. Accounting for a 10% non-response rate, we aimed to recruit 2151 participants pre- and post-campaign. Recruiting at least 2000 participants at each time point would provide 90% power to detect at least a 5% point difference on KAB questions pre- and post-campaign. Within the sample of participants who identified as a parent, recruiting at least 800 participants at each time point would provide 90% power to detect a 7% point difference on KAB questions pre- and post-campaign. 

### 2.4. Development and Implementation of the Consumer Awareness Campaign

**Figure 1:**
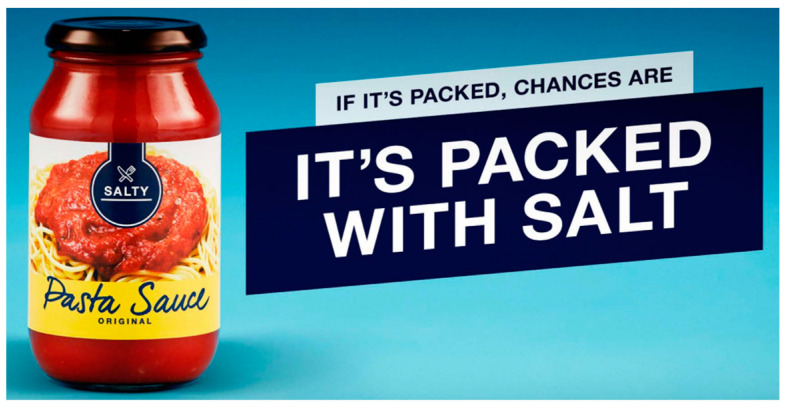
Advertisement used in the Unpack the Salt campaign.

The consumer awareness campaign was developed and implemented by the Heart Foundation (Victoria) and further details have been published elsewhere [20,39,40]. The selection of the target market (i.e., household food providers with children) was informed by evidence of (i) high salt intakes among children [11,41,42], (ii) recognized health benefits of early life salt reduction that track over the life course [43] and (iii) parents acting as nutritional ‘gate keepers’ to the family food environment [44]. There were two phases of the consumer awareness campaign: ‘Don’t Trust Your Taste Buds’ (June–July 2016) and ‘Unpack the Salt’ (August 2017 to April 2019). The campaign aimed to (i) raise awareness about health risks associated with excess salt intake during adulthood and childhood and how reducing salt intake can improve health, (ii) increase knowledge of daily salt intake recommendations and that 75% of salt in the Australian diet comes from processed foods (Figure 1) and (iii) educate consumers to read food labels to look for options containing less sodium and encourage the consumption of fresh, unprocessed foods that contain no added salt (e.g., fruits and vegetables) (https://youtu.be/t1EnYhYDlJA) (accessed on 10 July 2022). Key messages of the campaign mapped to evaluation questions are described in Supplementary Table S1. Advertising dissemination methods centered on paid digital and social media advertising. Table 1 shows an overview of campaign resources and reach of the campaign. 

### 2.5. Data Analysis

Survey data were analyzed using Stata/SE 15.1 (StataCorp LP, College Station, TX, USA). Data are reported as mean or % and SE or 95% CI. Post-stratification weights were created to weight the data to reflect the Victorian population of adults aged 18–65 years for sex and age group [24] and applied using the “pweight” functions in Stata. Unweighted results were primarily the same. Response options to KAB questionnaire items were generally dichotomized for analyses (e.g., strongly agree and agree vs. neither agree nor disagree, disagree and strongly disagree). Logistic regression analyses were used to assess differences in KAB responses pre- and post-campaign delivery in two groups (i) the target market of the campaign (e.g., parents with children <18 years) and (ii) the wider adult population (excluding parents of children <18 years) to determine if messages diffused beyond the target market. Pearson’s chi-squared tests and independent *t*-tests were used to ascertain whether demographic characteristics differed pre- and post-campaign. With the exceptions of age and sex, which were accounted for in post-stratification weighting, characteristics that differed (*p*-value < 0.20) were adjusted for in regression models. For parents this included country of birth (COB), educational attainment, diagnosis with a chronic condition, previously receiving information from a health professional to reduce salt intake and responsibility for household grocery shopping. For the wider adult sample this included COB, educational attainment, BMI (kg/m^2^), diagnosis with a chronic condition, previously receiving information from a health professional to reduce salt intake and responsibility for grocery shopping. Language spoken at home (*p*-value < 0.20) was not included as a covariate because it was associated with COB and COB was selected to indicate ethnicity. The collective diagnosis with a chronic condition was included as a covariate rather than individual conditions such as heart disease or stroke. For all models, the post-estimation “margins” command was used to derive adjusted proportions or adjusted salt intake for each time point. A *p*-value of <0.05 was considered significant. 

## 3. Results

### 3.1. Study Participants

**Figure 2:**
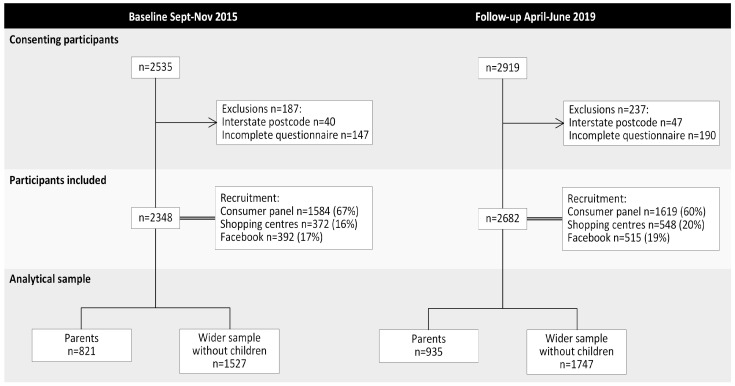
Flowchart of participation at baseline and follow-up.

In total, 5454 adults were invited to participate, and ineligible participants were excluded (pre *n* = 187; post *n* = 237) (Figure 2). Response rates for the shopping center intercept survey were 19% pre- and 11% post-campaign and the response rate for the online consumer research panel was 14% at both time points. It was not possible to determine the response rate for participants recruited via Facebook. At both time points, 35% of participants consisted of parents (pre *n* = 821 (709 parents, 112 caregivers); post *n* = 935 (846 parents, 89 caregivers)). The majority of parents were recruited via the consumer research panel (≈65%), with the remaining recruited via shopping centers (≈22%) and Facebook (≈13%). The sample of the wider adult population (excluding parents with children <18 years of age) pre- and post-campaign was 1527 and 1747, respectively. 

### 3.2. Parents

#### 3.2.1. Demographic Characteristics of Parents

Approximately 55% of parents were female with an average age of 41 (SD 10) years and about 40% were of a healthy weight (Table 2). Most parents (>69%) were born in Australia; however, post-campaign there was a greater percentage who were born outside of Australia and who spoke a language other than English at home. Approximately half of the sample had completed a University/tertiary qualification and about one-quarter reported that they had been diagnosed with a chronic condition. There were some differences in these characteristics pre- vs. post-campaign (Table 2). At both timepoints, about one-fifth of parents reported that they were aware of VicHealth’s initiative to reduce salt intake within the Victorian population (baseline 21% vs. follow-up 21%, *p* = 0.69).

#### 3.2.2. Difference in Salt-Related Knowledge, Attitudes and Behaviors among Parents following a Salt-Reduction Consumer Awareness Campaign

**Figure 3:**
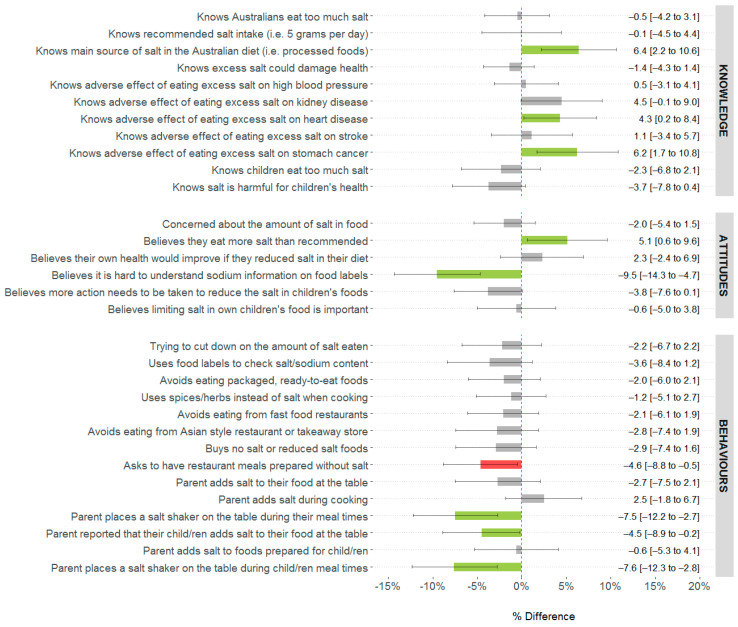
Difference in knowledge, attitudes and behaviors related to dietary salt among parents of children aged <18 years residing in the state of Victoria, Australia following a salt reduction consumer awareness campaign ^1, 2, 3^. ^1^. Analysis weighted to represent Victorian population (Census 2016) for age and sex [24]. ^2^. Data are mean [95%CI] and values are adjusted for country of birth, educational attainment, diagnosed with chronic condition, received advice from a health professional to reduce salt intake and responsibility for household grocery shopping. ^3^. Color legend for change in KABs: green bars represent a significant (*p* < 0.05) positive improvement; red bars represent a significant worsening; grey bars represent no change.

Following the campaign there were some small improvements in a few of the general salt-related knowledge (3/9) and attitude (2/4) items assessed. Specifically, the percentage of parents able to correctly identify processed foods as the main source of salt in the Australian diet increased by 6% and those aware of the link between excess salt and heart disease and stomach cancer both increased by 4–6% (all *p* < 0.05). There was a 10% reduction in parents who reported that it was difficult to understand sodium information on food labels and a 5% increase in parents who believed they ate more salt than was recommended (both *p* < 0.05). There was, however, no change in parents’ overall concern about the amount of salt in food (≈85% both time points) or in their belief that their own health would improve if they reduced their salt intake (≈45% both time points). There was no change in parents’ knowledge (0/2) or attitudes (0/2) that related to salt intake specifically during childhood (Figure 3, Supplementary Table S2). 

There was no change in the percentage of parents who reported trying to reduce their salt intake (≈42% both time points). There were some small improvements in discretionary salt use behaviors. Specifically, 5% fewer parents reported that their children added salt to their food at the table and 8% fewer reported that they placed a salt shaker on the table during meal times (both *p* < 0.05). Conversely, there was no change in parents’ reported use of cooking salt (to foods prepared for themselves or their children) or their own use of salt at the table. Overall, there was no change in other salt reduction behaviors (Figure 3, Supplementary Table S2).

### 3.3. Wider Adult Sample without Children Aged <18 Years

#### 3.3.1. Demographic Characteristics of Wider Adult Sample

At both time points approximately 50% of this sample were female with an average age of 41 years and 40% were of a healthy weight. Post-campaign there were more respondents who were born overseas, spoke a language other than English and had completed a University/tertiary qualification and fewer who reported a chronic condition or were receiving advice to reduce their sodium intake (Supplementary Table S3). Just under one-fifth of this group reported that they were aware of VicHealth’s initiative to reduce salt intake within the Victorian population (baseline 14% vs. follow-up 17%, *p* = 0.01). 

#### 3.3.2. Difference in Salt-Related Knowledge, Attitudes, Behaviors among Wider Adult Sample following a Salt Reduction Consumer Awareness Campaign

**Figure 4:**
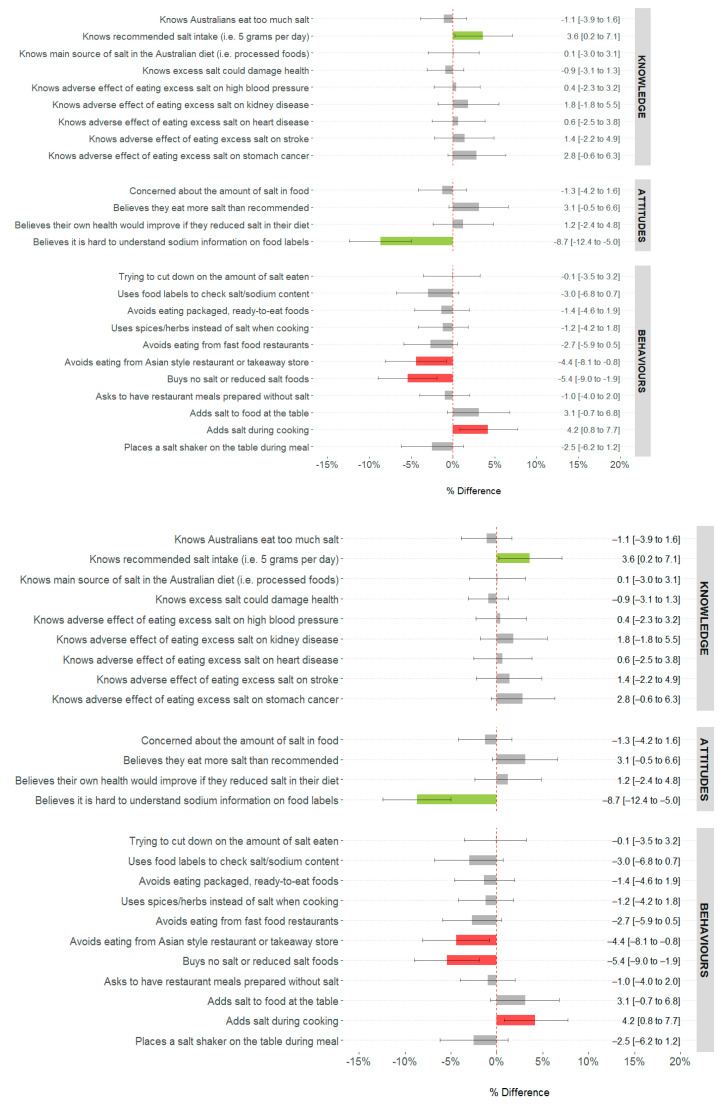
Difference in knowledge, attitudes and behaviors related to dietary salt among wider adult sample without children aged <18 years residing in the state of Victoria, Australia following a salt reduction consumer awareness campaign (*n* = 2900) ^1, 2, 3^. ^1^. Analysis weighted to represent Victorian population (Census 2016) for age and sex [24]. ^2^. Data are mean [95%CI] and values are adjusted for country of birth, educational attainment, BMI (kg/m^2^), diagnosed with chronic condition, received advice from a health professional to reduce salt intake and responsibility for grocery shopping. ^3^. Color legend for change in KABs: green bars represent a significant (*p* < 0.05) positive improvement; red bars represent a significant worsening; grey bars represent no change.

Overall, within this group, salt-related knowledge remained unchanged, the only exception was a small 4% increase in the percentage of adults who knew the recommended daily salt intake of 5 g/d (*p* < 0.05). Only one out of the four salt-related attitudes assessed shifted in a small positive direction, specifically post-campaign 9% fewer believed it was difficult to understand sodium information on food labels (*p* < 0.05) (Figure 4, Supplementary Table S4). There was no difference in the number of respondents who reported trying to limit their salt intake pre- vs. post-campaign (37% at both time points) and a number of salt-related reduction behaviors worsened post-campaign. Specifically, 4% more adults reported that they added salt during cooking and 4–5% fewer adults reported that they purchased reduced salt foods and avoided eating from Asian-style restaurants (*p* < 0.05). 

## 4. Discussion

A public awareness campaign “Unpack the Salt” was delivered within a multi-faceted state-wide population salt-reduction intervention within the state of Victoria, Australia from 2016 to 2019. Overall findings from this pre- vs post-campaign evaluation indicate the campaign was largely ineffective in shifting KABs related to salt intake within the community. Findings related to the target market of the campaign, i.e., parents, were somewhat more promising than the wider adult population; however, positive shifts in KABs, even among parents, were still limited and modest in size, e.g., only 25% of KAB indicators shifted in a positive direction, and improvements in the population ranged from 4–10% for knowledge and attitudes, and 5–8% for discretionary salt use behaviors. While it is unlikely that these small changes in KABs would translate into any meaningful reduction in salt consumption, the magnitude of change reported on KABs indicators is comparable to that achieved in previous health communication campaigns [45,46]. There was no evidence that campaign messages diffused to the wider adult population, as even fewer KABs indicators improved in this group and some salt-related behaviors actually worsened following the campaign. Overall these findings are similar to those previously reported in our 2018 mid-point evaluation of the consumer awareness campaign [22]. 

### 4.1. Key Findings and Implications for Messaging in Future Salt-Related KAB Campaigns

While there were a number of small shifts in knowledge and attitudes for both groups (e.g., parents and wider adult population) this did not appear to translate into substantial behavior change, with the only positive shift in behaviors related to some discretionary salt use practices among parents. Encouragingly, the largest improvement in knowledge among parents in our study related to one of the main campaign messages, which highlighted the ‘hidden’ salt in packaged and processed foods—that 75% of dietary salt comes from processed foods. Overall, parents and the wider adult population generally had good knowledge of high salt intakes within adults and the link with common health conditions. Recommendations for daily intakes of salt were not well understood by both groups, with most people thinking they ate about the right amount of salt; and less than half believing their own health would improve if they lowered their intake. These findings are consistent with previous samples of adults from high-income countries [47]. Given the overall disconnect between personal intakes and recommendations, it is unsurprising that there was no shift in the proportion of parents who reported taking action to lower their salt intake. Future salt reduction awareness campaigns may benefit by tailoring messages to bridge the gap between personal perceptions of salt intake, actual intake and health consequences. 

Although parents reported no change in their overall action to reduce salt in their own diet; small improvements were reported in discretionary salt use behaviors that related to their children (e.g., fewer children adding salt at the table and fewer parents placing a salt shaker on the table during meal times). These changes were consistent with those reported at the previous mid-point evaluation of the campaign [22] and directly align with the main messages of the campaign. On the campaign website among the four top tips to reduce salt intake, messages specifically related to table salt use were included e.g., “Stop adding salt to your food at the dinner table” and “Banish the salt shaker from your family’s table” [48]. Our findings suggest these messages may have reached some of the target market and the incorporation of simple messages targeting discretionary salt use may be useful as part of future salt reduction awareness campaigns. Whilst, reducing the amount of salt during cooking or at the table will not in itself substantially reduce salt intake in a population where most salt consumed comes from processed foods [49], it may represent one behavior that can easily be implemented by parents and included within wider reaching salt reduction strategies. 

### 4.2. Comparison to Past Consumer Awareness Campaigns 

This was the first salt awareness campaign to be implemented and evaluated within the Australian setting. Although our reported changes in KABs were modest; this is to be expected when considering outcomes achieved from previous health promotion awareness campaigns [45,46,50,51] and the relatively limited allocation of resources to the current campaign [21]. Internationally, one of the most notable consumer awareness campaigns targeting population salt reduction was conducted in the UK by the Food Standards Agency (FSA) between 2004 and 2009 [7]. This large-scale campaign included national coverage of messages across television, radio, press and digital advertising. The campaign was very well resourced (estimated cost GBP 41.6 million, not including staff costs (equivalent AUD 72.5 million)) [52,53] when compared to the current campaign undertaken in Australia (estimated cost approx. AUD 950,000, not including staff costs). Given this investment it is unsurprising that evaluations of the UK campaign indicated more promising changes in the populations’ KABs than that observed following the current campaign. For example, in the UK post-campaign, a much higher shift (+21% improvement) was reported in the population’s knowledge that processed food was the main source of salt in the diet (vs. +6% improvement in the current study). Importantly, and unlike the current campaign, in the UK there was also an observed effect on reported behavior, where significantly more adults (+26%) reported taking action to reduce salt in their diet [7,54]. Specifically, significantly fewer adults reported adding salt at the table (−17%) and during cooking (−6%) [55], estimates which are somewhat comparable to the reported shift downwards in salt use practices at the table among parents in the current study. While the UK campaign certainly showed promise in shifting consumers perceptions about salt in the diet during the first year, longer term follow-up of the campaign indicated these changes were not sustained and questioned the cost-effectiveness of this component of the wider population’s salt-reduction strategy [52,54]. Other countries which have shown some success in shifting population salt-related KAB include Samoa [56], Vietnam [57] and South Africa [58]. Similar to the situation in Australia, these campaigns were delivered within a wider population-based salt-reduction program and covered similar campaign messages. Overall findings from past evaluations of international salt awareness campaigns indicate greater improvements in KABs (e.g., positive shifts reported in ≥10% of the population) than that observed in the present study. A common feature across these previous campaigns was the use of paid television and radio advertisements, often at a national scale (e.g., UK and Vietnam) [7,57]. These dissemination methods likely enabled greater exposure to the campaign then the primarily paid digital advertising on social media used in the current campaign. This is supported by reported participant exposure within these past campaigns of 30–73% [56,58,59] compared to just ≈20% within the current study. 

Other locally delivered nutrition-related awareness campaigns have reported changes in population KABs that were more closely aligned to those observed in the current study. The Cancer Council and Heart Foundation’s “LiveLighter ‘Sugary Drinks’” campaign delivered in 2015 [51] and the Victorian state-government’s “2 Fruit ‘n’ 5 Veg Every Day” campaign delivered in the early 1990s [50] achieved changes in reported behavior [51] or reported behavior and knowledge [50] within the Victorian population (e.g., positive shifts reported in 3–9% of the population) that were comparable to the shifts reported in the current study. Both of these campaigns incorporated paid advertising over mass-media channels (e.g., TV and radio) and investment in advertising was greater than in the current campaign (e.g., “LiveLighter Sugary Drinks campaign” AUD 906,000; “2n5 Fruit and Vegetable campaign” AUD 376,544 in early 1990s, estimated equivalence of AUD 712,500 in 2018 versus current salt awareness campaign AUD 538,000). This likely contributed to the considerably wider reach achieved, with about half of surveyed respondents recalling these past campaigns. Collectively, when considering findings across these past campaigns as well as the limited resources available to the Victorian Salt Reduction Partnership, it may have been more beneficial for the current salt awareness campaign to consolidate advertising funds over a shorter time-period, e.g., 1 year, and across more diverse communication channels [46], including paid TV and radio advertising to maximize potential campaign reach, as opposed to a prolonged 4-year active campaign delivered primarily via online digital communications which relied solely on media advocacy methods to disseminate messages via mass media channels [20]. However, it is unclear if any shifts in KABs over a shorter evaluation period would be sustained over time. Findings from a consumer awareness campaign implemented earlier on in the 4-year salt-reduction initiative could have been used to support the development of other key intervention strategies, e.g., advocacy for government to set and strengthen policies related to salt-reduction and food industry action to lower the salt content of processed foods. 

### 4.3. Implications for Salt Reduction Initiatives

The consumer awareness campaign was included as one strategy to support population salt reduction within the multifaceted Victorian Salt Reduction Partnership intervention. This strategy as implemented was not enough to produce meaningful changes in the populations’ salt-related KABs. This may be in part be due to the overall low reach achieved with this primarily digitally delivered campaign. Previous consumer awareness campaigns, discussed above, which have incorporated a more diverse and wider media dissemination strategy, have reported higher participant exposure and more favorable changes in KAB. The World Health Organization’s SHAKE Technical Package for Salt Reduction advocates for the inclusion of consumer education as a means to place demand-side pressure on food manufacturers [6]; however, it is critical that this is incorporated with other wider reaching environmental strategies that seek to improve the food environment (e.g., sodium content targets for food reformulation). While national programs to reduce salt intake increased worldwide from 75 in 2014 to 96 in 2019, the number with consumer education campaigns has decreased from 71 to 50 in the same period [4]. This trend likely represents a shift towards initiatives that change the food environment, including introducing salt targets for food manufacturers, making food labeling easier for consumers to understand, food procurement policies and salt taxation, all of which increased over the same time period [4]. While the Victorian Salt Reduction Partnership did engage with food manufacturers and prompted some product changes, it lacked the federal regulatory levers needed for widespread and systematic change [21]. Moving forward in Australia, to reach the Government’s national target of at least a 30% reduction in population salt intake by 2030, there is an urgent and ongoing need for continued investment in a range of strategies that have been incorporated into international efforts to reduce population salt intake, namely food reformulation, food procurement policies, front-of-pack labeling, salt taxation and low-sodium salt substitutes [4,60]. 

### 4.4. Strengths and Limitations

A strength of this study was the large sample size and inclusion of age group and sex quota targets to help capture a representative sample of the Victorian population. The questionnaire was developed by experts working in the area of salt reduction and modeled on previous surveys. Our findings are subject to social desirability bias, for instance self-reporting of KABs that favor salt reduction, so it is acknowledged that results may not reflect actual KABs. Due to logistical constraints with the consumer research company, it was not possible to source independent samples post-campaign for participants recruited via the online consumer panel. Our analysis did not account for those participants (294/5030) who answered the survey at both time points. This approach introduces error related to inflated variability, which increases the likelihood of Type II error. However, as this applies to only 6% of respondents, impact is likely minimal. Finally, the repeated cross-sectional study design, means that trends in KABs were only assessed during the evaluation period, without a control group we cannot assess the effectiveness of the campaign on KABs, nor is it possible to discern potential secular trends on salt-related KABs external to the consumer awareness campaign. 

## 5. Conclusions

In this evaluation of a state-wide salt reduction consumer awareness campaign targeting parents residing in the state of Victoria, Australia we found minimal evidence that the campaign improved the groups’ salt-related KABs. Similarly, our findings indicated no diffusion of campaign messages within the wider adult population. As such it is questionable if the campaign would have made a meaningful contribution to the suite of strategies within the Victorian Salt Reduction Partnership seeking to reduce the population’s salt intake. One key message of the campaign that may have reached some parents related to removal of the salt shaker from the table and children adding less salt to their own food. Education-based strategies alone are unlikely to shift population salt intake downwards. Future salt-reduction initiatives that incorporate a consumer awareness campaign component should give careful consideration to associated costs of the campaign, duration, dissemination methods most likely to reach the target market, likely impact of the campaign on the population’s KAB and best methods to incorporate KAB evaluation findings within wider strategies and policies that seek to reduce salt consumption in the population.

## Figures and Tables

**Table 1:** Overview of campaign resources and dissemination methods [40].

Dissemination Method for Campaign Resources	Reach ^1^
Overall consumer awareness campaign	47.7 million people Including 3406 stories Equated to total cumulative advertising space rate $8,419,539
Campaign website (www.unpackthesalt.com.au) (accessed on 10 July 2022) Resources included on website: - Low salt recipes (*n* = 66) - Salt swap guides - Tips for reading food labels - Herb and spice cooking guide - Dietitian written blogs (*n* = 31)	Total visits during campaign *n* = 257,909
Campaign posters/billboards placed in shopping centers	
Heart Foundation social channels (e.g., Facebook, Youtube, Twitter, Instagram)	
Topical media stories on salt content of children’s foods during ‘back to school’ periods in 2018/19	
Media releases (*n* = 6) - These documented the salt content of six food categories known to contribute salt in the diet (cooking sauces, ready meals, dips and crackers, bread, processed meats, Asian-style sauces)	Lead to 759 media items across print and online news, radio, TV Cumulative audience reach (i.e., opportunities to see) for each media release ranged from 2.3 to 7.5 million Australians [20]

^1^ Data included where available.

**Table 2:** Demographic characteristics of parents with children aged <18 years pre- (*n* = 821) and post-campaign (*n* = 935) ^1^ showing unweighted numbers and percentages.

Characteristic	Pre-Campaign (2015)		Post-Campaign (2019)		*p*-Value ^2^
	*n*	%	*n*	%	
Sex					
Male	336	44.7	435	45.6	0.71
Female	485	55.3	500	54.4	
Age group (years)					
18–24	36	6.2	49	6.8	0.49
25–34	182	24.2	202	22.7	
35–44	310	37.2	322	36.2	
45–54	208	24.3	257	23.7	
55–65	85	8.1	105	10.6	
Country of Birth					
Australia	673	82.2	651	69.5	<0.001
United Kingdom	17	2.0	37	3.7	
New Zealand	9	1.1	21	2.2	
Other	112	13.4	213	23.2	
Don’t know/prefer not to answer	10	1.2	13	1.4	
Speaks a language other than English at home					
Yes	155	19.2	248	27.2	<0.001
No, English only	659	79.9	673	71.3	
Don’t know/prefer not to answer	7	0.9	14	1.6	
Educational attainment ^3^					
High (University/tertiary qualification)	362	44.9	528	57.1	<0.001
Mid (technical/trade certificate or diploma)	242	29.4	218	23.8	
Low (some or no level of high school education)	209	25.7	176	19.0	
Body mass index (mean (SE) kg/m^2^)	27.3 (0.2)	27.2 (0.2)	27.1 (0.2)	27.1 (0.2)	0.65
Weight category ^4^					
Underweight	15	2.2	19	2.4	0.45
Healthy weight	291	39.7	320	37.5	
Overweight	230	31.7	311	35.5	
Obese	199	26.5	213	24.6	
Diagnosed with a chronic condition					
Yes	233	28.4	208	21.9	0.001
No	575	70.0	721	77.4	
Don’t know/can’t recall	13	1.6	6	0.7	
Ever been diagnosed with or suffered from:					
Heart disease	40	5.6	19	2.1	<0.001
Stroke	37	5.0	12	1.4	<0.001
Heart attack	23	3.1	15	1.7	0.055
Other (please specify)	59	3.1	44	4.5	0.031
Don’t know/can’t recall	13	1.6	6	0.7	0.081
High blood pressure	173	5.6	143	15.1	0.001
Currently taking medication for blood pressure control					
Yes	115	66.4	96	67.3	0.87
No	58	33.6	47	32.7	
Ever received any advice from a doctor or health professional to reduce intake of salt/sodium and/or salty foods					
Yes	184	23.2	177	19.2	0.06
No	598	71.6	720	76.7	
Can’t recall	39	5.2	38	4.1	
Main person who does the grocery shopping in your household					
Yes	626	75.9	655	70.8	0.04
No	55	7.1	92	9.7	
No, I share the responsibility	140	17.0	188	19.5	

^1^. Demographic characteristics at baseline and follow-up weighted to represent Victorian population (Census 2016) for age and sex [24]. ^2^. *p*-value determined via Pearson’s chi-squared test or independent *t*-test. ^3^. Participants (pre *n* = 8; post *n* = 13) who responded “don’t know” or “prefer not to answer” for their highest level of education were excluded. ^4^. Participants (pre *n* = 86; post *n* = 72) who responded with missing data, “don’t know” or “prefer not to answer” for either height or weight were excluded.

## Data Availability

The mean data generated in the current study are included in this published article. The raw data used in this study cannot be made publicly available as participants did not provide consent for their data to be used for purposes other than described in the original study aims. The data that support the findings may be made available by the corresponding author for reasonable request upon approval by the Deakin University Human Research Ethics Committee.

## Supplementary Materials

The following supporting information can be downloaded at https://www.mdpi.com/article/10.3390/nu15040991/s1, Supplementary File S1: Salt-related knowledge, attitude and behavior questionnaire; Table S1: Key messages of the salt reduction consumer awareness campaign and corresponding KAB evaluation survey questions; Table S2: Difference in knowledge, attitudes and behaviors related to dietary salt among parents with children aged <18 years residing in the state of Victoria, Australia following a salt reduction consumer awareness campaign; Table S3: Demographic characteristics of the wider adult sample without children aged <18 years pre- (*n* = 1527) and post-campaign (*n* = 1747); Table S4: Difference in knowledge, attitudes and behaviors related to dietary salt among the wider adult sample without children aged <18 years residing in the state of Victoria, Australia following a salt reduction consumer awareness campaign (*n* = 2900). Source: [24,61,62].
