# Supplementary material for: Evaluation of a Salt-Reduction Consumer Awareness Campaign Targeted at Parents Residing in the State of Victoria, Australia
Source: Nutrients. 2023 Feb 16;15(4):991. doi: 10.3390/nu15040991 (PMC9964045; doi:10.3390/nu15040991)
Supplement: Supplementary file 1 [file nutrients-15-00991-s001.zip › Grimes Supplementary File_Final.pdf]

Supplementary tables

Supplementary Table S1. Key messages of the salt-reduction consumer awareness campaign and corresponding KAB evaluation survey questions

Campaign messages	Evaluation questions	Construct evaluated (KAB)[1]
Victorians eat almost twice as much as the recommended amount of salt	**Question:** In general, how much salt do you think Australians eat?<br>*Responses:* ***Far too much, too much****, just the right amount, too little, far too little, don't know*<br>**Question:** How do you think your daily salt intake compares to the amount of salt recommended by health professionals?<br>*Responses: I eat less salt than recommended; I eat about the right amount of salt; I eat more salt than recommended; I don't know*<br>**Question:** I am concerned about the amount of salt in food<br>*Responses: not at all concerned; not very concerned; somewhat concerned; very concerned; extremely concerned*	Knowledge (1 Q)<br>Attitude (2 Qs)
It is recommended that adults eat no more than 5 g/d of salt (1 teaspoon)	**Question:** Health professionals recommend that we should eat no more than a certain amount of salt each day. How much salt do you think this is?<br>*Responses: 3 grams (about ½ a teaspoon);* ***5 grams (about a teaspoon)****; 8 grams (about 1 and a ½ teaspoons; 10 grams (about 2 teaspoons); 15 grams (about 3 teaspoons); don't know*	Knowledge (1 Q)
75% of the salt in our diets comes from processed foods	**Question:** Which of the following do you think is the main source of salt in the Australian diet?<br>*Responses: salt added during cooking or at the table;* ***salt from processed foods such as breads, sausages and cheese****; salt from natural food sources; don't know*	Knowledge (1 Q)
Too much salt is bad for health and increases the risk of stroke, heart attack, kidney disease and stomach cancer	**Question:** Do you think that eating too much salt could damage your health?<br>*Responses:* ***Yes;*** *no; don't know*<br>**Question:** Which, if any, of the following conditions do you think is linked to eating too much salt? High blood pressure; kidney disease; heart disease/heart attack; stroke; stomach cancer<br>*Responses:* ***Yes;*** *no; don't know*	Knowledge (2 Qs, 1 Q included 5 items)
Changing your diet can lower your risk of heart attack and stroke.	**Question:** Do you think that eating too much salt could damage your health?<br>*Responses: Yes; no; don't know*<br>**Question:** I am concerned about the amount of salt in food<br>*Responses: not at all concerned; not very concerned; somewhat concerned; very concerned; extremely concerned*	Attitude (2 Qs)
Promote adoption of low salt behaviours to reduce salt consumption e.g. resources available via campaign website and promoted via digital advertising such as low salt recipes, salt swaps guide, tips for reading food labels, herb and spice cooking guide, blogs written by a dietitian with tips for reducing salt in the diet.	**Question:** Are you trying to cut down on the amount of salt you eat?<br>*Responses: Yes; no; don't know*<br>**Question:** Below are some common ways to lower the amount of salt in your diet. In the past month, to what extent have you personally done any of the following?<br>- Looked at a food label to check the salt/sodium content of a food item<br>- Purchased foods labelled "no added salt", "salt reduced" or "reduced sodium"<br>- - Used spices/herbs instead of salt when cooking<br>- Avoided eating packaged, ready-to-eat foods	Behaviours (5 Qs, 1 Q included 7 items)

	- Avoided eating food from fast food restaurants (e.g. McDonalds, KFC, Pizza Hut)<br>- Avoided eating food from an Asian style restaurant or takeaway store (e.g. Chinese, Thai, Indian)<br>- When eating out, asked to have your meal prepared without salt<br>*Responses: never do this; rarely do this; sometimes do this; often do this; always do this; does not apply to me*<br>**Question:** How often do you add salt to your food at the table?<br>**Question:** In the food you eat at home, how often is salt added during cooking?<br>**Question:** Do you place a salt shaker on your table at meal times?<br>*Responses: always, often, sometimes, rarely, never, don't know*	
	**Question:** It is hard to understand sodium information displayed on food labels<br>*Responses: 5-point Likert scale strongly disagree-strongly agree*	Attitudes (1 Q)
**Only evaluated in parents/caregivers**		
Too much salt during childhood can lead to a lifetime of health risks	**Question:** In the long term eating too much salt during childhood may have harmful effects on children's health<br>*Responses: **strongly agree, agree**, neither agree nor disagree, disagree, strongly disagree*	Knowledge (1 Q)
Children eat more salt than is recommended	**Question:** In general how much salt do you think Australian children eat?<br>*Responses: **far too much, too much**, just the right amount, too little, far too little, don't know*	Knowledge (1 Q)
Increase concern about their family's salt intake	**Question:** Limiting the amount of salt my child/or children eat/s is important to me<br>*Responses: 5-point Likert scale, strongly agree-strongly disagree*	Attitude (1 Q)
Support for reduction of salt levels in processed foods	**Question:** Medical research suggests that most Australian children eat more salt than is good for them. Do you think more action needs to be taken to reduce the salt in foods targeted at children.<br>*Responses: 5-point Likert scale, strongly disagree-strongly agree*	Attitude (1 Q)
Promote adoption of low salt behaviours to reduce families salt consumption	**Question:** Do you add salt to foods you prepare for your child or children?<br>*Responses: always; often; sometimes; rarely; never; not applicable, I do not prepare food for my child*<br>**Question:** Do you place a salt shaker on your table at meal times?<br>*Responses: always; often; sometimes; rarely; never; don't know*<br>**Question:** Does your child or children add salt to their food at the table?<br>*Responses: always; often; sometimes; rarely; never; don't know*	Behaviours (3 Qs)

[1] For knowledge questions, bolded responses represent correct answers

Supplementary Table S2. Difference in knowledge, attitudes and behaviours related to dietary salt among parents with children aged <18 years residing in the state of Victoria, Australia following a salt-reduction consumer awareness campaign (n=1735)[1, 2, 3]

Item	Pre: 2015 n=813 %	Post: 2019 n=922 %	% difference (Post-pre)	p-value[4]
**Knowledge**				
Knows Australians eat too much salt	83.2%	82.7%	-0.5 (-4.2, 3.1)	0.77
Knows recommended salt intake e.g. 5 grams/d	29.7%	29.6%	-0.1 (-4.5, 4.4)	0.97
Knows main source of salt in Australian diet comes from processed food i.e. bread, sausages, cheese	72.0%	78.4%	**6.4 (2.2, 10.6)**	**0.003**
Knows excess salt could damage health	91.1%	89.7%	-1.4 (-4.3, 1.4)	0.32
Knows adverse effect of eating excess salt on:				
High blood pressure	83.7%	84.2%	0.5 (-3.1, 4.1)	0.81
Kidney disease	62.7%	67.1%	4.5 (-0.1, 9.0)	0.06
Heart disease	75.6%	79.8%	**4.3 (0.2, 8.4)**	**0.04**
Stroke	67.0%	68.1%	1.1 (-3.4, 5.7)	0.63
Stomach cancer	31.0%	37.3%	**6.2 (1.7, 10.8)**	**0.008**
Knows children eat too much salt	71.8%	69.4%	-2.3 (-6.8, 2.1)	0.30
Knows salt is harmful for children's health	78.2%	74.5%	-3.7 (-7.8, 0.4)	0.08
**Attitudes**				
Concerned about amount of salt in food[5]	86.7%	84.7%	-2.0 (-5.4, 1.5)	0.26
Believes they eat more salt than recommended	28.3%	33.4%	**5.1 (0.6, 9.6)**	**0.03**
Believes their own health would improve if they reduced salt in their diet[6]	44.3%	46.6%	2.3 (-2.4, 6.9)	0.33
Believes it is hard to understand sodium information on food labels[6]	51.4%	41.9%	**-9.5 (-14.3, -4.7)**	**<0.001**
Believes more action needs to be taken to reduce the salt in children's foods[6]	81.5%	77.8%	-3.8 (-7.6, 0.1)	0.06
Believes limiting salt in own children's food is important[6]	70.5%	69.9%	-0.6 (-5.0, 3.8)	0.79
**Behaviors**				
Trying to cut down on the amount of salt eaten	43.6%	41.3%	-2.2 (-6.7, 2.2)	0.32
Uses food labels to check salt/sodium content[7]	58.2%	54.6%	-3.6 (-8.4, 1.2)	0.14
Avoids eating packaged, ready-to-eat foods[7]	77.6%	75.7%	-2.0 (-6.0, 2.1)	0.35
Uses spices/herbs instead of salt when cooking[7]	81.0%	79.8%	-1.2 (-5.1, 2.7)	0.55
Avoids eating from fast food restaurants[7]	79.4%	77.3%	-2.1 (-6.1, 1.9)	0.30
Avoids eating from Asian style restaurant or takeaway store[7]	66.8%	64.0%	-2.8 (-7.4, 1.9)	0.24
Buys no salt or reduced salt foods[7]	70.3%	67.4%	-2.9 (-7.4, 1.6)	0.21
Asks to have restaurant meals prepared without salt[7]	29.8%	25.1%	**-4.6 (-8.8, -0.5)**	**0.03**

Supplementary tables

**Parents'/caregivers' own discretionary salt use behaviours**				
Adds salt to their own food at the table[8]	54.1%	51.4%	-2.7 (-7.5, 2.1)	0.26
Adds salt during cooking[8]	69.4%	71.9%	2.5 (-1.8, 6.7)	0.25
Places a salt shaker on table at meal times[8]	48.9%	41.4%	**-7.5 (-12.2, -2.7)**	**0.002**
**Child/ren's discretionary salt use behaviours**				
Child/ren adds salt to food at the table[8]	31.7%	27.2%	**-4.5 (-8.9, -0.2)**	**0.04**
Adds salt to foods prepared for your children[8]	47.9%	47.3%	-0.6 (-5.3, 4.1)	0.80
Places a salt shaker on table at children's meal times[8]	44.7%	37.1%	**-7.6 (-12.3, -2.8)**	**0.002**

[1] Analysis weighted to represent Victorian population (Census 2016) for age and sex [1]

[2] Values adjusted for COB, educational attainment, diagnosed with chronic condition, received advice from a health professional to reduce salt intake, responsible for household grocery shopping

[3] Adjusted analysis excludes those with missing information for SES n=8 at baseline and n=13 at follow-up

[4] p-value determined by logistic regression model

[5] Responses grouped as 'very, extremely and somewhat concerned' vs. 'not very, not at all concerned'

[6] Responses grouped as 'strongly agree, agree' vs. 'strongly disagree, disagree, neither agree nor disagree'

[7] Responses grouped as 'always do this, often do this, sometimes do this' vs. 'rarely do this, never do this'; 'does not apply to me' excluded from analysis

[8] Responses grouped as 'always, often, sometimes' vs. 'rarely, never'; 'don't know' responses excluded

Bolded data indicate difference was statistically significant from pre to post-campaign p<0.05

Supplementary tables

Supplementary Table S3. Demographic characteristics of the wider adult sample without children aged <18 years pre (n=1527) and post-campaign (n=1747) showing unweighted numbers and percentages

Characteristic	Pre (2015)		Post (2019)		p-value[2]	Victorian population (%)[3]
	n	%[1]	n	%[1]		
**Sex**						
Male	683	51.4	843	51.0	0.83	49.1
Female	844	48.6	904	49.0		50.9
**Age group (years)**						
18-24	208	19.6	261	19.2	0.85	14.9
25-34	320	23.1	409	24.0		23.6
35-44	203	13.0	232	13.6		21.3
45-54	299	18.7	387	19.0		20.7
55-65	497	25.7	458	24.1		19.5
**Country of Birth**						
Australia	1206	79.3	1292	73.5	**0.003**	
United Kingdom	66	3.7	91	4.9		
New Zealand	18	1.1	36	2.0		
Other	223	14.7	314	18.7		
Don't know/prefer not to answer	14	1.1	14	0.9		
**Speaks a language other than English at home**						
Yes	249	17.5	335	20.3	0.19	
No, English only	1267	81.6	1397	78.8		
Don't know/prefer not to answer	11	0.9	15	0.9		
**Educational attainment**						
High (University/tertiary qualification)	461	30.8	478	28.3	**0.001**	28.1[4]
Mid (technical/trade certificate or diploma)	641	42.6	841	49.0		27.0
Low (some or no level of high school education)	414	26.6	399	22.7		42.9
**Body mass index (mean (SE) kg/m^2)**	26.9 (0.2)	26.5 (0.2)	27.1 (0.2)	26.8 (0.2)	0.17	
**Weight category[5]**						
Underweight	52	4.4	50	3.5	0.30	2.3[6]
Healthy weight	534	41.9	635	42.0		37.7
Overweight	447	31.6	469	29.9		30.6
Obese	327	22.1	406	24.6		19.1
**Diagnosed with a chronic condition**						

Yes	458	27.0	435	24.1	0.19
No	1049	71.6	1288	74.4	
Don't know/can't recall	20	1.4	24	1.4	
**Ever been diagnosed with or suffered from:**					
Heart disease	62	3.8	58	3.3	0.42
Stroke	30	2.0	19	1.2	0.067
Heart attack	31	1.8	26	1.6	0.55
Other (please specify)	135	8.0	131	7.3	0.45
Don't know/can't recall	22	1.5	24	1.4	0.84
**High blood pressure**	331	19.2	298	16.3	**0.025**
Currently taking medication for blood pressure control	265				
Yes	265	78.9	235	78.9	0.99
No	66	21.1	63	21.1	
**Ever received any advice from your doctor or a health professional to reduce your intake of salt/sodium and/or salty foods**					
Yes	295	18.8	255	14.6	**0.007**
No	1153	76.1	1401	80.2	
Can't recall	79	5.2	91	5.3	
**Main person who does the grocery shopping in your household**					
Yes	993	61.5	1111	61.9	0.13
No	176	14.2	187	11.8	
No, I share the responsibility	358	24.4	449	26.3	

[1] Demographic characteristics at baseline and follow-up weighted to represent Victorian population (Census 2016) for age and sex[1]

[2] p-value determined via Pearson's chi-squared test and for BMI (kg/M^2) linear regression, all based on weighted data.

[3] Except where otherwise indicated data taken from the 2016 Victorian Census and reflects the percentage of adults aged 18–65 years residing in Victoria[1]

[4] Data taken from the 2016 Survey of Education and Work and includes information on educational attainment in Victorian adults aged 15–74 years[2]. Participants (pre n=11; post n=29) who responded "don't know" or "prefer not to answer" for their highest level of education were excluded

[5] Participants (pre n=167; post n=187) who responded with missing data or "don't know" or "prefer not to answer" for either height or weight were excluded

[6] Data taken from the 2016 Victorian Population Health Survey 2016, estimates based on self-reported height and weight[3]

Supplementary Table S4. Difference in knowledge, attitudes and behaviours related to dietary salt among the wider adult sample without children aged <18 years residing in the state of Victoria, Australia following a salt-reduction consumer awareness campaign (n=2900)[1, 2, 3]

Item	Pre: 2015 n=1356 %	Post: 2019 n=1544 %	% difference (Post - Pre)	p-value[4]
**Knowledge**				
Knows Australians eat too much salt	85.2%	84.1%	-1.1 (-3.9, 1.6)	0.42
Knows recommended salt intake e.g. 5 grams/d	28.2%	31.8%	**3.6 (0.2, 7.1)**	**0.039**
Knows main source of salt in Australian diet comes from processed food i.e. bread, sausages, cheese	79.3%	79.4%	0.1 (-3, 3.1)	0.96
Knows excess salt could damage health	91.8%	90.9%	-0.9 (-3.1, 1.3)	0.44
Knows adverse effect of eating excess salt on:				
High blood pressure	84.5%	84.9%	0.4 (-2.3, 3.2)	0.76
Kidney disease	63%	64.9%	1.8 (-1.8, 5.5)	0.33
Heart disease	78.5%	79.2%	0.6 (-2.5, 3.8)	0.70
Stroke	65.1%	66.5%	1.4 (-2.2, 4.9)	0.46
Stomach cancer	28.8%	31.6%	2.8 (-0.6, 6.3)	0.11
**Attitudes**				
Concerned about amount of salt in food [5]	82.7%	81.4%	-1.3 (-4.2, 1.6)	0.38
Believes they eat more salt than recommended	31.1%	34.2%	3.1 (-0.5, 6.6)	0.088
Believes their own health would improve if they reduced salt in their diet[6]	40.9%	42.1%	1.2 (-2.4, 4.8)	0.52
Believes it is hard to understand sodium information on food labels[6]	44.7%	36%	**-8.7 (-12.4, -5)**	**<0.001**
**Behaviors**				
Trying to cut down on the amount of salt eaten	37.2%	37.1%	-0.1 (-3.5, 3.2)	0.93
Uses food labels to check salt/sodium content [7]	55.8%	52.8%	-3 (-6.8, 0.7)	0.11
Avoids eating packaged, ready-to-eat foods [7]	76.8%	75.4%	-1.4 (-4.6, 1.9)	0.40
Uses spices/herbs instead of salt when cooking [7]	81.5%	80.3%	-1.2 (-4.2, 1.8)	0.45
Avoids eating from fast food restaurants [7]	78.9%	76.2%	-2.7 (-5.9, 0.5)	0.098
Avoids eating from Asian style restaurant or takeaway store [7]	64.9%	60.5%	**-4.4 (-8.1, -0.8)**	**0.018**
Buys no salt or reduced salt foods [7]	69.4%	64%	**-5.4 (-9, -1.9)**	**0.003**
Asks to have restaurant meals prepared without salt [7]	19.9%	18.9%	-1 (-4, 2)	0.51
Adds salt to food at the table[8]	46%	49%	3.1 (-0.7, 6.8)	0.11
Adds salt during cooking[8]	63.5%	67.7%	**4.2 (0.8, 7.7)**	**0.015**
Places a salt shaker on the table during meal[8]	47.2%	44.7%	-2.5 (-6.2, 1.2)	0.19

[1] Analysis weighted to represent Victorian population (Census 2016) for age and sex [1]

[2] Values adjusted for COB, educational attainment, BMI (kg/m^2), diagnosed with chronic condition, received advice from a health professional to reduce salt intake, responsibility for grocery shopping
[3] Adjusted analysis excludes those with missing information for SES n=11 at baseline and n=29 at follow-up and for BMI n=167 at baseline and n=187 at follow-up
[4] p-value determined by logistic regression model
[5] Responses grouped as 'very, extremely and somewhat concerned' vs. 'not very, not at all concerned'
[6] Responses grouped as 'strongly agree, agree' vs. 'strongly disagree, disagree, neither agree nor disagree'
[7] Responses grouped as 'always do this, often do this, sometimes do this' vs. 'rarely do this, never do this'; 'does not apply to me' excluded from analysis
[8] Responses grouped as 'always, often, sometimes' vs. 'rarely, never'; 'don't know' responses excluded
Bolded data indicate difference was statistically significant from pre to post-campaign p<0.05

References

1. Australian Bureau of Statistics. 2016 Census Community Profiles, Victoria. Available online: https://quickstats.censusdata.abs.gov.au/census_services/getproduct/census/2016/communityprofile/2?opendocument. (Accessed on 6th June 2022).
2. Australian Bureau of Statistics. 6227.0 - Education and Work, Australia, May 2016. Available online: http://www.abs.gov.au/AUSSTATS/abs@.nsf/DetailsPage/6227.0May%202016?OpenDocument. (Accessed on 20 Nov 2022).
3. Department of Health and Human Services. *Victorian Population Health Survey 2016: Selected survey findings, State of Victoria, Melbourne;* Melbourne, Victoria, 2018.
